# Associations of physical partner violence and sexual violence victimization on health risk behaviours and mental health among university students from 25 countries

**DOI:** 10.1186/s12889-020-09064-y

**Published:** 2020-07-02

**Authors:** Supa Pengpid, Karl Peltzer

**Affiliations:** 1 ASEAN Institute for Health Development, Mahidol University, Salaya, Phutthamonthon, Nakhon Pathom, Thailand; 2 Department of Research Administration and Development, University of Limpopo, Turfloop, South Africa; 3 Department of Psychology, University of the Free State, Bloemfontein, South Africa

**Keywords:** Physical partner violence victimization, Sexual violence victimization, Risk behaviour, Mental health, University students, Multi-country

## Abstract

**Background:**

The study aimed to investigate the associations between physical partner violence victimization (IPV) and/or sexual violence victimization and various health risk behaviours and mental health in university students in 25 countries.

**Methods:**

Using a cross-sectional study design, 18,335 university students with a median age of 20 years from 25 countries in Africa, the Americas and Asia, replied to self-reported measures of interpersonal violence, health compromising behaviours, mental health measures and protective factors.

**Results:**

In adjusted logistic regression analysis, physical IPV and/or sexual violence victimization was associated, among men and/or among women, with sexual risk behaviours (multiple sexual partners, alcohol use in the context of sex, diagnosed with HIV and pregnancy), violence related behaviour (in a physical fight and carrying a weapon), poor mental health (depression, loneliness, post-traumatic stress disorder, sleeping problem and short sleep), addictive behaviour (binge drinking, tobacco and drug use), and other health risk behaviour (skipping breakfast and frequent salt intake).

**Conclusions:**

We found evidence that physical IPV and/or sexual violence victimization among female and/or male university students was associated with 4 of 5 sexual risk behaviours, 2 violence related behaviours, 5 of 5 poor mental health indicators, 3 of 3 addictive behaviours and 2 of 7 other health risk behaviours.

## Background

Intimate partner violence (IPV) and other violence among adolescents and emerging adults is being increasingly recognized as common and having a wide range of health, behavioural and well-being consequences [1]. Various studies have identified that a history physical IPV and/or sexual violence victimization among male and/or female adolescents or emerging adults is associated with sexual risk behaviours, violence related behaviours, poor mental health, substance use, other health risk behaviours, poor health status and poor academic performance. Sexual risk behaviour among adolescents and emerging adults may include, multiple sexual partners [2–4], unprotected sex [5–9], pregnancy [4–7, 10], and sexually transmitted infections including HIV [5, 10–13].

Violence-related behaviours among adolescents and emerging adults may include in a physical fight [2, 14], carried a weapon [2], handgun access [15], and injury [5, 10]. Poor mental health as a consequence of physical IPV and/or sexual violence victimization among adolescents and emerging adults may include depression [5, 9, 11, 12, 16–20], posttraumatic stress [5, 11, 12, 16], anxiety [5, 9, 16, 19], psychological distress [8, 21], suicidal behaviour [2, 14, 17, 20], sleep disorders [5, 12, 13], and disordered eating [5, 10]. Several studies found that a history of physical IPV and/or sexual violence victimization among adolescents and emerging adults increased the odds for substance use, such as alcohol use or binge drinking [2, 5, 9, 10, 13], tobacco use [5, 6], drug use [2, 5, 6, 9, 10, 14], and substance use at last sex [3]. Other health risk behaviours include physical inactivity [5], eating unhealthy foods [15], such as inadequate fruit and vegetable intake [22], and not wearing a seatbelt [15]. Moreover, some studies found as a consequence of physical IPV and/or sexual violence victimization a poor self-rated health status [21, 23], poor quality of life [19], and poorer educational outcomes [9, 20].

Most studies reviewed above investigated associations between physical IPV and/or sexual violence victimization with single or a few negative outcomes. However, there is a lack of studies examining multiple outcomes, such as sexual risk behaviours, violence related behaviours, poor mental health, substance use, other health risk behaviours, poor health status and poor academic performance in the same study population. In particular, information is scarce on multiple consequences of physical IPV and/or sexual violence victimization in university students in low- and middle-income countries. “While there has been much empirical research on adult dating violence, only recently has research began to also focus on young adult dating violence in general.” [24] Therefore, the study aimed to assess the associations of physical IPV and/or sexual violence victimization with various health risk indicators and mental health among university students in 25 countries in the Americas, Africa and Asia.

## Methods

### Sample and procedure

The cross-sectional study comprised 18,335 college or university students with a median age of 20 years (interquartile range = 3 years) with complete physical IPV and/or sexual violence victimization data from 25 countries in Southeast Asia: Indonesia, Laos, Philippines, Singapore and Thailand; South Asia and China: India, Pakistan and China; North Africa and Central Asia: Egypt, Tunisia, Turkey, Russia and Kyrgyzstan; the Americas: Barbados, Columbia, Grenada, Jamaica and Venezuela and in sub-Saharan Africa: Cameroon, Ivory Coast, Madagascar, Mauritius, Namibia, Nigeria and South Africa. The study was initiated through personal, academic contacts of the principal investigators; thus, in each study country one or two universities were purposefully selected, [25] targeting 400 male and 400 female undergraduate university students aged 16–30 years. “The participants were identified using a quasi-random selection process, which entailed randomly selecting one department from each University faculty, and a random selection was then made from an ordered list of all undergraduate courses offered within the selected department” [25] “Trained research assistants then described the study to students within the selected undergraduate class to recruit participants. The inclusion criterion was being present in class at the time of recruitment.” [25]. The consent form included a written justification of the study and contact details of the local principal investigator were provided in order to respond to any questions or personal concerns. Trained research assistants administered the paper-based self-administered questionnaire and made sure that each student had privacy when filling in the questionnaire. The consent forms and the questionnaires (without identifying information) were collected separately and placed in different boxes in the front of the rooms. “Informed consent was attained from all participating students, and ethics approvals were obtained from all participating universities. Participation rates were in most countries over 90%” [26].

“For the population survey, the expected frequency of 50% (maximum possible percentage of students with positive and negative health behaviors), design effect 1 (in calculation used given the ratio of the actual variance, under the sampling method, to the variance computed under the assumption of simple random sampling), confidence limited 5%, cluster 1, form this confidence limited 5% key in to calculating formula, will get outcome of sample size calculated for seven confidence levels, the researchers chose sample size for confidence of 99%, the minimum sample size is 663.To prevent incomplete data, the sample size was increased to 800 (400 male, 400 female).” [27].

### Measures

*Physical IPV and sexual violence victimization* were sourced from two questions: “1) Have you ever been hit by a sexual partner? and 2) Have you ever been forced to have sex?” (yes, no) [28].

*Socio-demographic* information comprised gender, age, subjective wealth status and country income.

*Social support* was sourced from three questions of the “Social Support Questionnaire” [29] (Cronbach’s alpha 0.94).

*Physical childhood abuse* was assessed with item: “Have you ever been physically abused as a child” (yes, no) [30].

*Sexual risk behaviour* included “two or more sexual partners in the past 12 months”, “alcohol use in the context of sex in the past 3 months”, “history of having been diagnosed with a sexually transmitted infection (STI)”, “diagnosed with HIV” and “ever made someone pregnant/been pregnant” [31].

#### Violence related behaviour

*Having been in a physical fight* was defined as “having been involved in a physical fight one or more times in the past 12 months” [32].

*Weapon carrying* was defined as “carrying a weapon such as a gun, knife, or club” on one or more days in the past month [33].

*Injury* was defined as “serious injury when it makes you miss at least one full day of usual activities (such as college, sports, studies or a job) or requires treatment by a doctor or nurse” in the past 12 month [32].

#### Mental health

*The “Centres for Epidemiologic Studies Depression Scale (CES-D-10)”* measured with 15 or more scores severe depressive symptoms [34]. (Cronbach’s alpha = 0.76).

*Loneliness* was defined as most (5–7 days) feeling lonely in the past week [34].

*“Post-traumatic stress disorder (PTSD)”* was measured with “Breslau’s 7-item screener” [35].

*Sleeping problem* was defined as “an extreme/cannot do problem with sleeping, such as falling asleep, waking up frequently during the night, or waking up too early in the morning.” [36].

*Sleep duration*: “On average, how many hours of sleep do you get in a 24 h period?” “Responses were divided into three categories: short sleep (≤6 h), reference category (7–8 h), and long sleep (≥9 h)” [37].

#### Addictive behaviour

*Binge drinking (past-month)* was assessed with the item, “How often do you have (for men) five or more and (for women) four or more drinks on one occasion?” [38].

*Tobacco use* was defined as currently using “tobacco products (cigarettes, snuff, chewing tobacco, cigars, etc.)” [39].

*Illicit drug use* was defined as “10 or more times having taken any drugs other than those prescribed by health care providers in the past 12 months.” [40]

#### Other health risk behaviour

*Physical activity* was measured using the “International Physical Activity Questionnaire (IPAQ) short form” [41], and physical activity levels were defined following IPAQ guidelines [42].

*Breakfast consumption* was measured with the item, “How often do you eat breakfast?” (“Almost every day, sometimes, rarely or never”) [43]. Skipping breakfast was defined as sometimes, rarely or never having breakfast.

*Fruit and vegetable intake* was measured with two questions, 1) “How many servings [80 grams] of fruit do you eat on a typical day?” and 2)“How many servings [80 g] of vegetables do you eat on a typical day?” [44]. Inadequate fruit and vegetable intake was defined as “less than 5 servings a day” [45].

*Adding salt to food* was defined as usually adding salt to food (options: “usually, sometimes, occasionally, never”) [43].

*Seatbelt use* was measured with the item, “When driving or riding in the front seat of a car do you wear a seat belt?” (“1 = all of the time, 2 = some of the time, 3 = never and 4 = I don’t ride in cars”) [43].

*Self-rated health status* was assessed by a single item, “In general, would you say that your health is … Excellent, Very good, Good, Fair or Poor” [43].

*Poor academic performance* was self-reported as “not satisfactory academic performance.” [46].

### Data analysis

Data analysis was conducted with “STATA software version 15.0 (Stata Corporation, College Station, TX, USA)”. Unadjusted and multivariable (adjusted for relevant confounders age, study year, subjective economic status, country, social support, and exposure to physical and/or sexual violence in childhood) logistic regression was used to assess the associations between physical and/or sexual IPV victimization and dependent variables (5 sexual risk behaviours, 3 violence related behaviours, 5 poor mental health indicators, 3 addictive behaviours, 5 other health risk behaviours, and academic performance). Due to the clustered nature of the data, country was entered in the survey command. Missing data (< 8% on any variable) were excluded from the analysis. *P* values < 0.05 were considered significant.

## Results

### Descriptive results

The sample consisted of 18,335 university students (median age 20 years, interquartile range 3 years) from 25 countries, 59.0% were females and 41.0% males, 52.6% rated their economic status as high, 57.3% had high social support, 46.0% were residing in low- or lower middle-income countries. A history of child physical abuse was reported by 4.8, and 8.0% had been exposed to physical IPV and/or sexual violence victimization.

In terms of sexual risk behaviour, 19.2% of students reported to have had multiple sexual partners in the past 12 months, 14.1% had used alcohol in the context of sex, 5.7% had a history of a sexually transmitted infection, 0.6% had been diagnosed with HIV and 8.1% had been or made someone pregnant. Regarding violence related behaviour, 12.7% of students had been involved in physical fighting in the past year, 6.0% had carried a weapon in th past 30 days, and 24.5% had sustained a serious injury in the past year. Mental health indicators showed that 13.0% of students had depression symptoms, 10.3% loneliness, 19.7% PTSD, 10.2% sleeping problems and 39.7% short sleep. In terms of addictive behaviour, 12.5% of students were current binge drinkers, 12.4% current tobacco users and 3.7% frequent drug users. Regarding other health risk behaviours, 42.2% of students were physically inactive, 46.6% skipped breakfast, 81.3% had inadequate fruit and vegetable intake, 40.0% had salt usually and 53.9% had not always been a seatbelt. Almost one in ten students (8.8%) rated their health status as poor, and 6.1% rated their academic performance as poor.

### Associations between physical IPV and/or sexual violence victimization and outcome variables

In adjusted logistic regression analyses among both sexes, physical IPV and/or sexual violence victimization was associated with sexual risk behaviour (having had multiple sexual partners, alcohol use in the context of sex, diagnosed with HIV and pregnancy), violence related behaviour (been in a physical fight and carried a weapon). Regarding poor mental health, among women physical IPV and/or sexual violence victimization was associated depression, lonelinss, PTSD, sleeping problem and short sleep, while among men only PTSD and sleeping problem were associated. In terms of addictive behaviour, among both sexes physical IPV and/or sexual violence victimization was associated with tobacco use, while among men with drug use and among women with binge drinking. Among men tobacco and drug use was associated. Moreover, among both sexes, physical IPV and/or sexual violence victimization was associated with skipping breakfast and frequent salt intake (see Table 1).

**Table 1:** Associations of physical IPV and/or sexual victimization on risk behaviours and mental health

Variable (prevalence)		All	Male	Female
		OR (95% CI)	OR (95% CI)	OR (95% CI)
**Sexual risk behaviour**				
Two or more sexual partners in past year (19.2%)	UOR	3.65 (1.90, 7.02)***	3.41 (2.01, 5.77)***	4.08 (1.54, 10.85)***
	AOR^a^	3.81 (2.28, 6.36)***	3.74 (2.40, 5.83)***	4.11 (2.05, 8.22)***
Alcohol use in the context of sex (14.1%)	UOR	3.26 (2.16, 4.93)***	2.74 (1.74, 4.30)***	4.00 (2.50, 6.38)***
	AOR^a^	3.14 (2.14, 4.60)***	3.01 (2.05, 4.40)***	3.21 (2.00, 5.18)***
History of sexually transmitted infection (5.7%)	UOR	1.90 (0.58, 6.19)	1.89 (0.67, 5.31)	1.92 (0.52, 7.01)
Diagnosed with HIV (0.6%)	UOR	23.08 (11.69, 46.34)***	28.04 (11.33, 71.62)***	19.04 (9.72, 37.03)***
	AOR^a^	11.43 (5.73, 22.82)***	15.73 (6.96, 33.51)***	8.87 (3.99, 19.72)***
Pregnancy (8.1%)	UOR	5.74 (4.01, 8.21)***	3.63 (2.79, 5.76)***	8.25 (5.09, 13.37)***
	AOR^a^	5.49 (3.92, 7.70)***	3.50 (2.38, 5.13)***	8.13 (4.90, 13.49)***
**Violence related behaviour**				
Was in a physical fight in the past 12 month (12.7%)	UOR	3.14 (2.16, 4.56)***	3.22 (2.17, 5.76)***	2.87 (2.03, 4.06)***
	AOR^a^	2.52 (1.68, 3.77)***	2.85 (1.97, 4.13)***	1.97 (1.38, 2.81)***
Weapon carrying in the past 30 days (6.0%)	UOR	2.57 (1.85, 3.56)***	2.56 (1.80, 3.65)***	2.29 (1.66, 3.18)***
	AOR^a^	2.14 (1.64, 2.78)***	2.34 (1.77, 3.09)***	1.72 (1.23, 2.43)**
Injury in the past year (24.5%)	UOR	1.28 (0.96, 1.72)	1.29 (0.91, 1.83)	1.20 (0.86, 1.69)
**Mental health**				
Depression severe (13.0%)	UOR	1.92 (1.50, 2.45)***	1.46 (1.04, 2.03)*	2.35 (1.75, 3.15)***
	AOR^b^	1.40 (1.13, 1.74)**	1.16 (0.86, 1.56)	1.63 (1.18, 2.25)**
Loneliness (10.3%)	UOR	1.62 (1.20, 2.19)**	1.25 (0.78, 2.01)	1.97 (1.35, 2.87)***
	AOR^c^	1.41 (1.06, 1.86)*	1.12 (0.72 1.74)	1.69 (1.13, 2.13)**
Post-traumatic stress disorder (19.7%)	UOR	2.47 (1.90, 3.22)***	1.97 (1.47, 2.64)***	2.99 (2.21, 4.03)***
	AOR^d^	1.92 (1.52, 2.42)***	1.66 (1.30, 2.13)***	2.18 (1.57, 3.02)***
Sleeping problem (10.2%)	UOR	1.83 (1.40, 2.39)***	1.90 (1.35, 2.68)***	1.79 (1.26, 2.55)**
	AOR^c^	1.71 (1.34, 219)***	1.80 (1.31, 2.49)***	1.65 (1.16, 2.33)**
Short sleep (39.7%)	UOR	1.33 (1.09, 1.62)**	1.26 (1.00,1.59)*	1.38 (1.08, 1.75)*
	AOR^c^	1.28 (1.03, 1.58)*	1.19 (0.92, 1.54)	1.35 (1.06, 1.71)*
**Addictive behaviour**				
Current binge drinking (12.5%)	UOR	1.91 (1.28, 2.84)**	1.38 (0.82, 2.32)	2.61 (1.82, 3.75)**
	AOR^e^	1.88 (1.34, 2.64)***	1.38 (0.87, 2.19)	2.56 (1.84, 3.55)***
Current tobacco use (12.4%)	UOR	3.13 (2.12, 4.63)***	2.92 (2.01, 4.23)**	3.34 (2.22, 5.04)***
	AOR^a^	2.57 (1.75, 3.77)***	2.60 (1.84, 3.67)***	2.45 (1.70, 3.54)***
Drug use (10 or more times in past 12 months) (3.7%)	UOR	2.04 (1.27, 3.26)**	1.98 (1.01, 3.90)*	2.04 (1.02, 4.09)*
	AOR^a^	1.72 (1.11, 2.67)*	1.80 (1.00, 3.31)*	1.56 (0.81, 3.04)
**Other health risk behaviour**				
Physical inactivity (42.2%)	UOR	0.81 (0.56, 1.18)	0.83 (0.58, 1.20)	0.84 (0.55, 1.29)
Skipping breakfast (46.6%)	UOR	1.33 (1.12, 1.58)**	1.42 (1.18, 1.71)***	1.27 (0.98, 1.64)
	AOR^a^	1.20 (1.04, 1.40)*	1.42 (1.19, 1.69)***	1.06 (0.85, 1.33
Inadequate fruit and vegetable intake (81.3%)	UOR	0.91 (0.71,1.18)	0.79 (0.64, 0.98)*	1.05 (0.70, 1.58)
Salt usually (40.0%)	UOR	1.44 (1.04, 2.00)*	1.43 (0.99, 2.07)	1.47 (1.05, 2.05)*
	AOR^a^	1.46 (1.14, 1.88)**	1.43 (1.05, 1.93)*	1.53 (1.19, 1.98)**
Not always wearing a seatbelt (53.9%)	UOR	0.88 (0.63, 1.24)	0.88 (0.60, 1.30)	0.88 (0.59, 1.29)
**Subjective health (poor)** (8.8%)	UOR	1.42 (0.89, 2.28)	1.56 (0.94, 2.68)	1.07 (0.71, 1.60)
**Academic performance (poor)** (6.1%)	UOR	0.97 (0.69, 1.35)	0.96 (0.68, 1.34)	0.92 (0.54, 1.57)

*UOR* unadjusted odds ratio, *AOR* adjusted odds ratio; ***P < 0.001; **P < 0.01; *P < 0.05; ^a^Model 1: Adjusted for age, sex, study year, social support, family income or wealth, country, exposure to physical violence in childhood, binge drinking, depression and PTSD; ^b^Adjusted for all variables in model 1, except for depression; ^c^Adjusted for all variales in model 1, expect for depression and PTSD; ^d^Adjusted for all variales in model 1, except for PTSD; ^e^Adjusted for all variables in model 1, except for binge drinking

### Associations of physical IPV and/or sexual violence victimization with risk behaviours by region

Dividing our study population into five regions (Americas, sub-Saharan Africa, North Africa and Central Asia, South Asia and China and Southeast Asia), results show that physical IPV and/or sexual violence victimization was associated with multiple sexual partners and pregnancy in three regions (Americas, sub-Saharan Africa and South Asia and China, and Americas, sub-Saharan Africa and North Africa and Central Asia, respectively). Further, physical IPV and/or sexual violence victimization was associated with alcohol use in the context of sex and weapon carrying in four regions (sub-Saharan Africa, North Africa & Central Asia, South Asia and China and Southeast Asia). Associations with involvement in physical fighting was found in two regions (sub-Saharan Africa and North Africa & Central Asia). An association with skipping breakfast was only found in the sub-Saharan African region, and a positive association with salt intake was found in the Americas and a negative association with salt intake in South Asia and China (see Table 2).

**Table 2:** Associations of physical IPV and/or sexual victimization with risk behaviours by region

Variable	Region (prevalence of outcome variable)	AOR^a^ (95% CI)
**Risk behaviour**		
Two or more sexual partners in past year	Americas (26.1%)	2.21 (1.46, 3.36)***
	Sub-Saharan Africa (24.2%)	3.21 (2.47, 4.19)***
	North Africa & Central Asia (36.8%)	2.32 (0.89, 6.04)
	South Asia and China (1.5%)	12.86 (4.29, 38.54)***
	Southeast Asia (3.5%)	2.53 (0.50, 12.84)
Alcohol use in the context of sex	Americas (25.2%)	1.60 (0.94, 2.73)
	Sub-Saharan Africa (14.1%)	2.62 (1.63, 4.23)***
	North Africa & Central Asia (9.9%)	4.38 (3.37, 5.70)***
	South Asia and China (2.0%)	24.92 (2.75, 225.53)**
	Southeast Asia (9.7%)	4.68 (1.61, 13.60)**
Pregnancy	Americas (12.4%)	3.51 (2.42, 5.10)***
	Sub-Saharan Africa (16.2%)	3.34 (2.16, 5.18)***
	North Africa & Central Asia (5.0%)	7.95 (4.04, 8.76)***
	South Asia and China (1.8%)	5.28 (0.81, 4.10)
	Southeast Asia (2.8%)	2.84 (0.71, 11.44)
Was in a physical fight in the past 12 month	Americas (10.7%)	1.36 (0.94, 1.99)
	Sub-Saharan Africa (14.4%)	2.12 (1.26, 2.58)**
	North Africa & Central Asia (18.3%)	3.49 (2.72, 4.46)***
	South Asia and China (12.1%)	1.97 (0.93, 4.19)
	Southeast Asia (7.2%)	3.17 (0.96, 10.37)
Weapon carrying in the past 30 days	Americas (6.2%)	1.39 (0.95, 2.02)
	Sub-Saharan Africa (5.7%)	2.10 (1.51, 2.93)***
	North Africa & Central Asia (9.1%)	2.14 (1.56, 2.95)***
	South Asia and China (5.1%)	2.49 (1.50, 4.13)***
	Southeast Asia (3.5%)	5.31 (3.86, 7.28)***
Skipping breakfast	Americas (43.8%)	1.44 (0.98, 2.10)
	Sub-Saharan Africa (49.9%)	1.21 (1.01, 1.44)*
	North Africa & Central Asia (45.9%)	1.13 (0.84, 1.51)
	South Asia and China (37.2%)	1.29 (0.77, 2.16)
	Southeast Asia (51.9%)	1.06 (0.62, 1.83)
Salt usually	Americas (27.3%)	1.33 (1.01, 1.76)*
	Sub-Saharan Africa (49.9%)	1.26 (0.86, 1.84)
	North Africa & Central Asia (52.9%)	1.25 (0.90, 1.74)
	South Asia and China (38.4%)	0.58 (0.32, 0.93)*
	Southeast Asia (27.1%)	0.92 (0.42, 1.99)

*AOR* adjusted odds ratio; ***P < 0.001; **P < 0.01; *P < 0.05; ^a^Adjusted for age, sex, study year, social support, family income or wealth, country, exposure to physical violence in childhood, binge drinking (if not outcome variable), depression (if not outcome variable) and PTSD (if not outcome variable)

### Associations of physical IPV and/or sexual violence victimization with mental health and substance use by region

Physical IPV and/or sexual violence victimization was associated with current binge drinking and current tobacco use in all five study regions. Associations with PTSD and sleeping problem were found in all five regions except for North Africa and Central Asia for PTSD and Southeast Asia for sleeping problem. Associations with depression were found for two regions (Americas and sub-Saharan Africa) and associtions with short sleep were found for th Americas and South Asia and China. Finally an association with loneliness was only found in students from sub-Saharan Africa and with drug use in students from Southeast Asia (see Table 3).

**Table 3:** Associations of physical IPV and/or sexual victimization with mental health and substance use by region

Variable	Region (prevalence of outcome variable)	AOR^a^ (95% CI)
**Mental health**		
Depression severe	Americas (12.8%)	2.04 (1.63, 2.55)***
	Sub-Saharan Africa (12.8%)	1.32 (1.12, 1.55)***
	North Africa & Central Asia (16.2%)	1.06 (0.82, 1.37)
	South Asia and China (17.9%)	1.45 (0.90, 2.35)
	Southeast Asia (6.8%)	1.16 (0.74, 1.83)
Loneliness	Americas (10.6%)	1.30 (0.94, 1.80)
	Sub-Saharan Africa (10.8%)	1.52 (1.17, 1.96)**
	North Africa & Central Asia (12.3%)	1.03 (0.75, 1.41)
	South Asia and China (12.8%)	1.43 (0.89, 2.30)
	Southeast Asia (5.1%)	1.75 (0.55, 5.63
Post-traumatic stress disorder	Americas (18.3%)	2.68 (2.00, 3.60)***
	Sub-Saharan Africa (20.2%)	2.22 (2.76, 2.80)***
	North Africa & Central Asia (24.7%)	1.32 (0.81, 2.14)
	South Asia and China (19.4%)	2.73 (1.53, 4.94)***
	Southeast Asia (16.4%)	2.80 (2.19, 3.57)***
Sleeping problem	Americas (10.1%)	1.58 (1.27, 1.96)***
	Sub-Saharan Africa (11.1%)	1.97 (1.58, 2.45)***
	North Africa & Central Asia (9.9%)	1.56 (1.24, 1.97)***
	South Asia and China (8.4%)	1.82 (11.0, 2.99)*
	Southeast Asia (10.8%)	1.06 (0.36, 3.05)
Short sleep	Americas (42.1%)	1.34 (1.00, 1.80)*
	Sub-Saharan Africa (42.5%)	1.05 (0.82, 1.33)
	North Africa & Central Asia (35.8%)	1.15 (0.75, 1.76)
	South Asia and China (28.9%)	2.82 (1.18, 6.76)*
	Southeast Asia (45.1%)	1.49 (0.68, 3.28)
**Addictive behaviour**		
Current binge drinking	Americas (27.5%)	1.27 (1.02, 1.59)*
	Sub-Saharan Africa (11.6%)	1.45 (1.02, 2.07)*
	North Africa & Central Asia (10.6%)	1.94 (1.38, 2.71)***
	South Asia and China (2.7%)	10.41 (9.01, 13.54)***
	Southeast Asia (10.6%)	2.93 (2.05, 4.17)***
Current tobacco use	Americas (10.0%)	1.41 (1.00, 2.00)*
	Sub-Saharan Africa (12.6%)	2.45 (1.52, 3.97)***
	North Africa & Central Asia (23.4%)	2.87 (2.33, 3.54)***
	South Asia and China (7.5%)	6.88 (3.11, 15.20)***
	Southeast Asia (6.0%)	2.30 (1.03, 5.16)*
Drug use (10 or more times in past 12 months)	Americas (4.4%)	0.86 (0.51, 1.44)
	Sub-Saharan Africa (7.3%)	1.46 (0.92, 2.31)
	North Africa & Central Asia (2.5%)	1.99 (0.85, 4.64)
	South Asia and China (0.9%)	0.88 (0.18, 4.16)
	Southeast Asia (4.2%)	3.42 (2.36, 4.96)***

*AOR* adjusted odds ratio; ***P < 0.001; **P < 0.01; *P < 0.05; ^a^Adjusted for age, sex, study year, social support, family income or wealth, country, exposure to physical violence in childhood, binge drinking (if not outcome variable), depression (if not outcome variable) and PTSD (if not outcome variable)

## Discussion

Consistent with findings of various previous studies [2–5, 10–12], this study found among men and women that physical IPV and/or sexual violence victimization was associated with sexual risk behaviour (having had multiple sexual partners, alcohol use in the context of sex, diagnosed with HIV and pregnancy), and violence related behaviour (been in a physical fight and carried a weapon). In agreement with previous findings [2, 5, 6, 8–13, 16–20], physical IPV and/or sexual violence victimization was overall and among women associated with all 5 mental health indicators (depression, loneliness, PTSD, sleeping problem and short sleep) and among men 2 poor mental health indicators (PTSD and sleeping problem), and overall victimization was associated with 3 addictive behaviours (alcohol, tobacco and drug use), among women 2 addictive behaviours (binge drinking and tobacco use) and among men 2 addictive behaviours (tobacco use and drug use).

The study also found some evidence that physical IPV and/or sexual violence victimization was associated other health risk behaviours, such as an unhealthy diet (skipping breakfast and frequent salt intake), as found in a previous study [15]. Contrary to some previous investigations [5, 15, 19–23], this study did not find an association between physical IPV and/or sexual violence victimization and physical inactivity, inadequate fruit and vegetable intake, not always wearing a seatbelt, poor self-rated health status, and poorer educational outcomes. In addition, some studies found an association between physical IPV and injury [5, 10], while this study did not find this association. This may be explained by failing to assess injurious consequences of physical IPV in this study.

Our results demonstrate the wide range of associations between physical IPV and/or sexual violence victimization and multiple outcomes, such as sexual risk behaviours, violence related behaviours, poor mental health, substance use and other health risk behaviours, with similar patterns observed for male and female students as well as across the five study regions. Generally, both men and women responded to victimization with internalizing symptoms (PTSD and sleeping problem) and externalizing symptoms (sexual risk behaviour, violence related behaviour and tobacco use). While some of the sex differences may be explained by women responding more likely than men in terms of internalizing symptoms and men more likely than women with externalizing symptoms [17]. For example, women had significantly more internalizing symptoms in terms of depressive symptoms, loneliness and short sleep, than men, while men had significantly more externalizing symptoms in the case of frequent drug use than women. However, mixed evidence for this was found in the case of substance use, where women in this study more likely than men responded with the externalizing symptoms of binge drinking.

In a stratified analysis by study region, associations of physical IPV and sexual violence victimization with the various significant outcome variables in the global analysis were only significant for the majority of the five study regions in case of four risk behaviours (multiple sexual partners, alcohol use in the context of alcohol use, pregnancy and weapon carrying), while involvement in physical fighting, skipping breakfast and taking salt usually was only significant in one or two study regions. One explanation for the non-significant association in some study regions may be attributed to the low prevalence of the outcome variable in specific study regions, e.g., pregnancy and frequent drug use in the South Asia and China region, and depression and loneliness in the Southeast Asia region.

Some of the mechanisms by which violence victimization leads to various consequences have been suggested. For example, trauma theory suggests that violence victims experience their violence victimization as traumatic leading to post-traumatic stress [47, 48]. Students experiencing adverse outcomes may appraise victimization as psychologically stress ful, and then use unhealthy coping processes to deal with this demand [49, 50]. Some of these unhealthy coping behaviours may include substance use, violence related behaviour or sexual risk behaviour.

Primary prevention (stopping physical IPV and sexual violence victimization before occurring) is the preferred intervention to prevent various risk behaviours (sexual, violence, substance use and others) and poor mental health. Primary prevention is likely to reduce victimization and various risk behaviours, including poor mental health [14]. In addition, secondary prevention is indicated in limiting or preventing negative consequences of victimization, in particular by addressing identified health risk behaviours and poor mental health indicators. Therefore, the assessment of frequent health risk behaviours and poor mental health indicators among victims of physical IPV and/or sexual violence and interventions targeting to ameliorate these behaviours and incidators will help in reducing short- and long-term health and social consequences [14]. Programmes targeting the prevention of physical IPV and sexual violence victimization among university students should include multiple risk behaviours, including sexual risk behaviour, violence related activities, poor mental health and substance use. In a systematic review, including interventions for dating and intimate partner violence among young persons (15–30 years), Jennings et al. [24] found that “when considered as a whole, the interventions tended mainly to have a mixed impact with respect to reducing dating/intimate partner violence between treatment and control groups, with most evidence pointing toward promising short-term effects that decayed over time.”.

### Study limitations

The study survey was limited to a cross-sectional design, participants attending university and most of the questionnaire administered was by self-report. The questionnaire administered was by self-report, and therefore some of the responses may have been biased; especially sexual risk behaviours and physical IPV and sexual violence victimization among both males and females, may have been underreported. The survey data were limited to cross-sectional. For example, it remains unclear what mechanism or chronology was involved in relation to physical IPV and/or sexual violence victimization and pregnancy, e.g., if physical IPV victimization led to the inability to use condoms, if the abusive partner actively prevented contraception use or if the abused feared using condoms. The study only assessed physical IPV and sexual violence victimization with yes/no response options and did not measure the frequency and severity of victimization, which should be included in future studies with more comprehensive measures. For some variables the confidence intervals were large, e.g. for having been diagnosed with HIV and for some types sexual risk behaviour in students from South Asia and China. This is a further limitation to the interpretation of these specific results, probably due to the low occurrence of the risk behaviour.

## Conclusion

This investigation added evidence for a large university population from 25 countries that physical IPV and/or sexual violence victimization was among male and/or female students associated with 4 of 5 sexual risk behaviours (multiple sexual partners, alcohol use in the context of sex, diagnosed with HIV and pregnancy), 2 violence related behaviours (been in a physical fight and carrying a weapon), 5 of 5 poor mental health indicators (depression, loneliness, PTSD, sleeping problem and short sleep), 3 of 3 addictive behaviours (binge drinking, tobacco and drug use) and 2 of 7 other health risk behaviours (skipping breakfast and frequent salt intake). Interventions to prevent physical IPV and sexual violence victimization in this population may include multiple levels, including university- and community-based interventions.

## Data Availability

The data for the current study will not be shared publicly as participants were informed at the time of providing consent that only researchers involved in the project would have access to the information they provided.

## Abbreviations

IPV: Intimate partner violence
IPAQ: International physical activity questionnaire (IPAQ)
PTSD: Posttraumatic stress disorder
